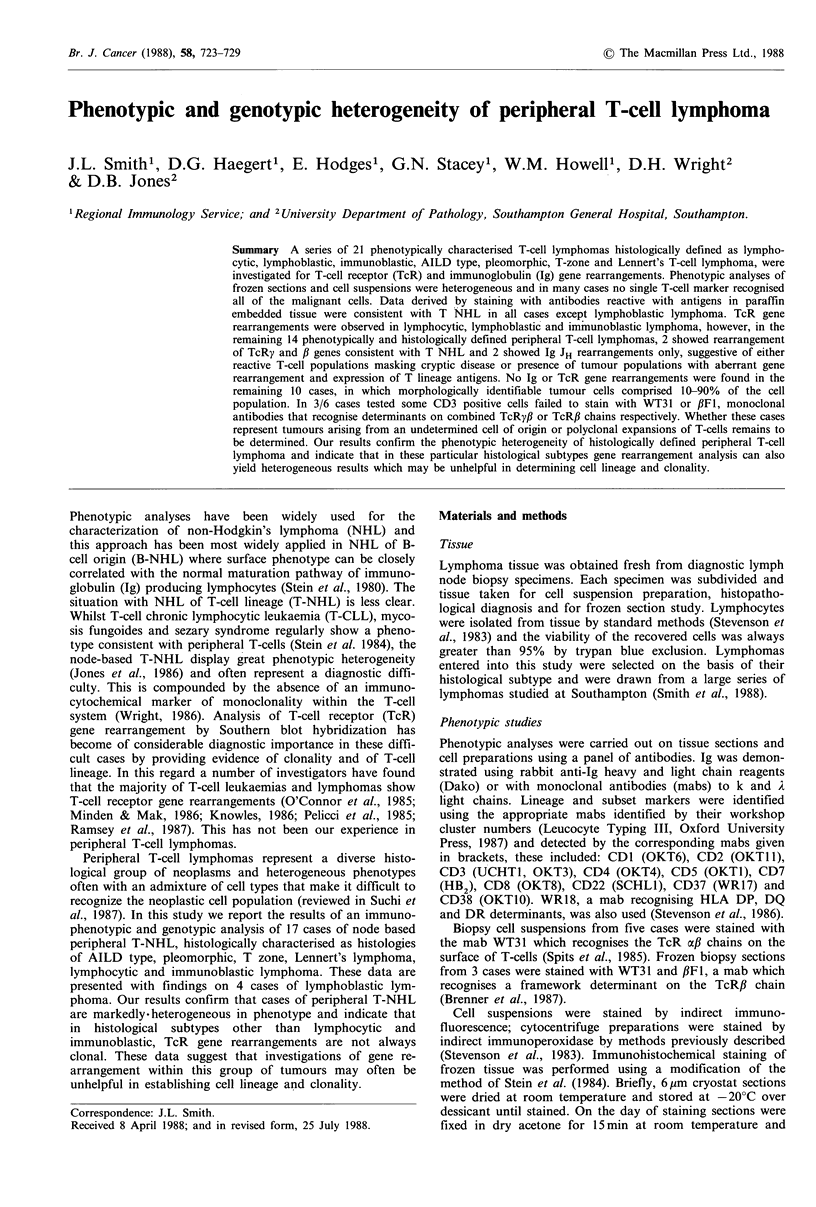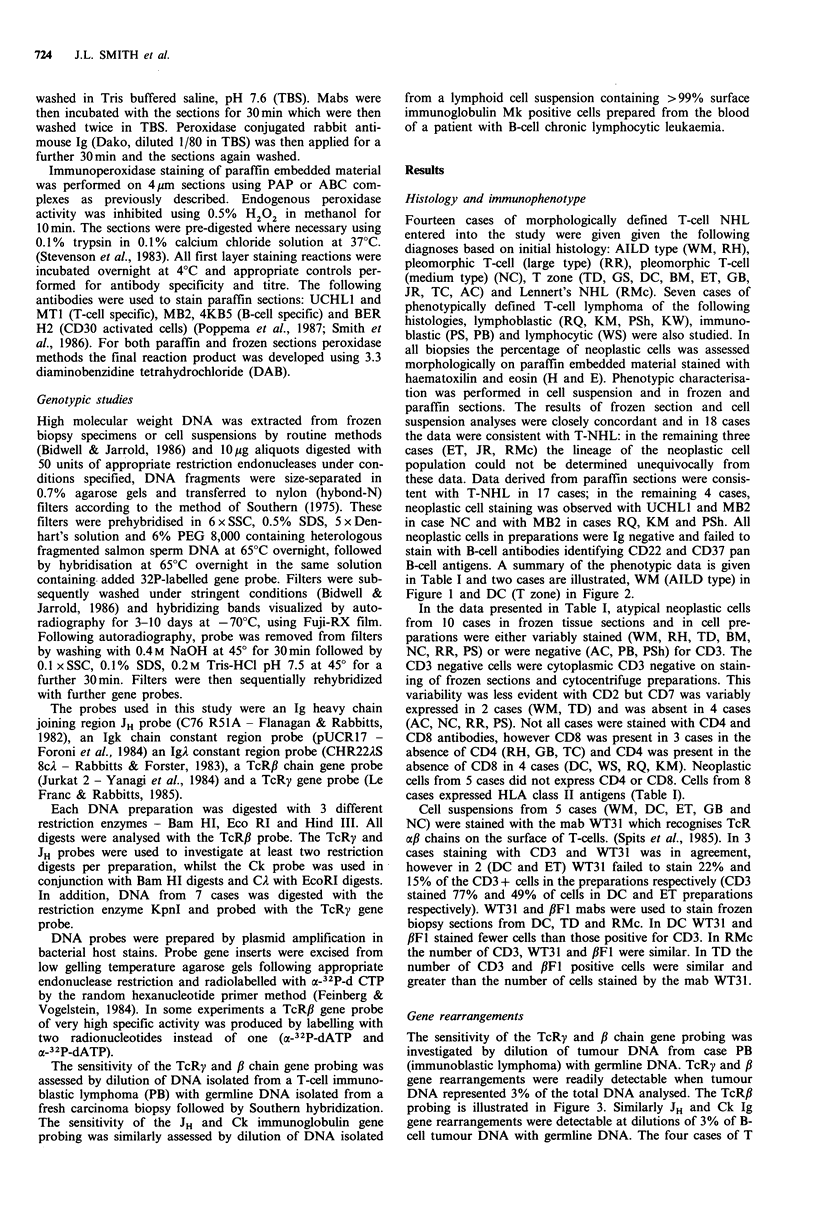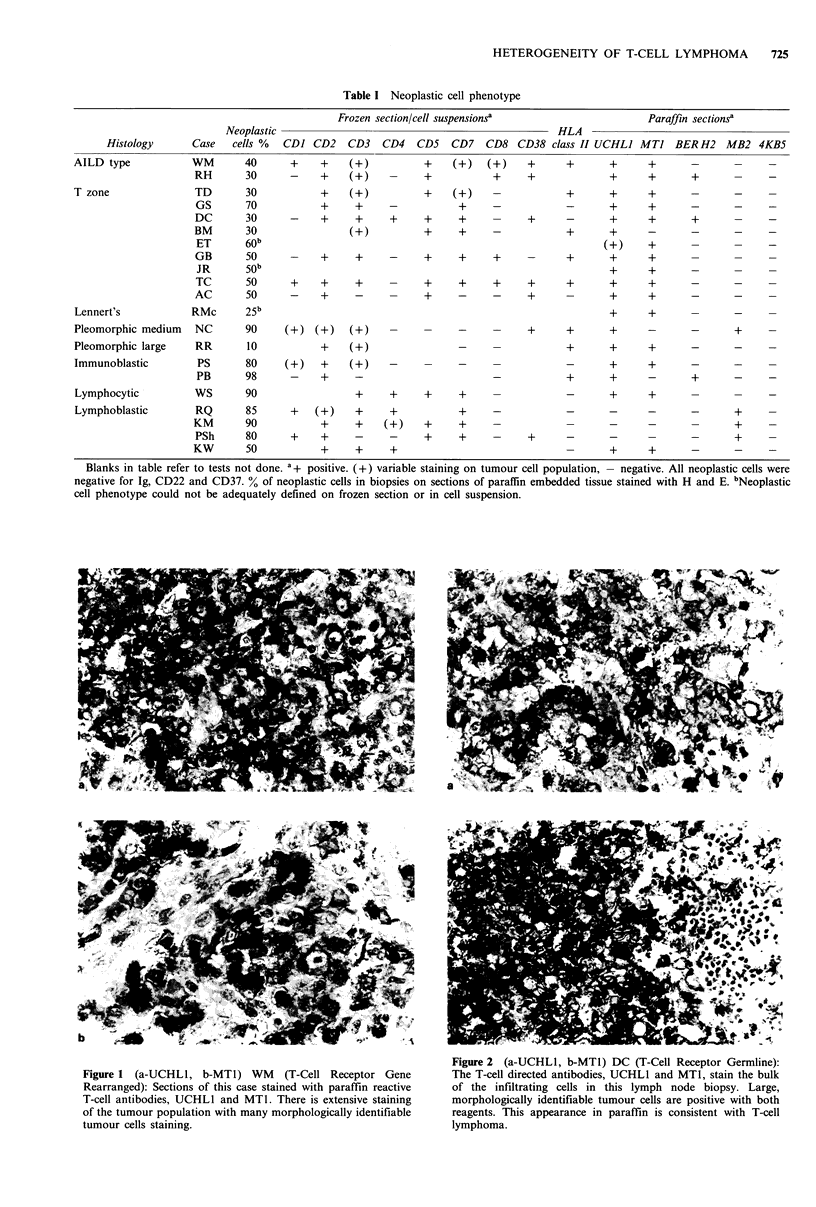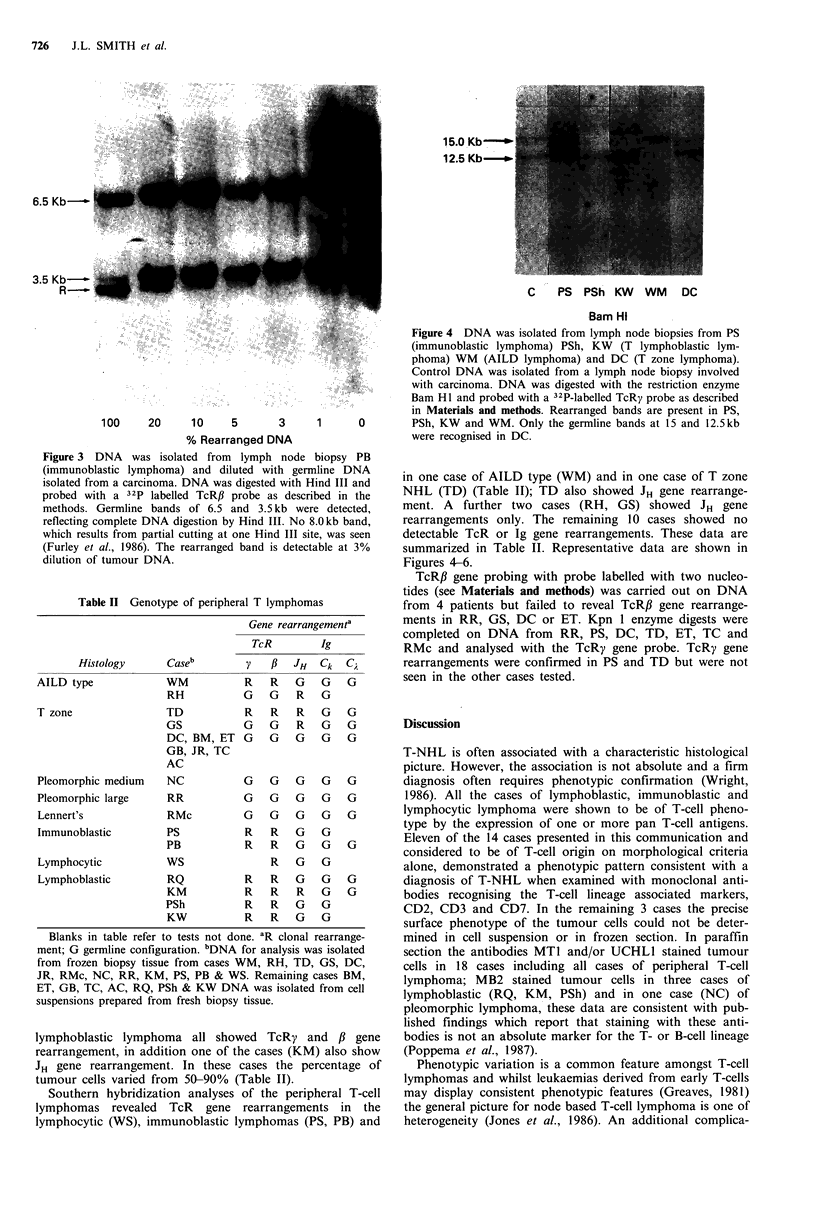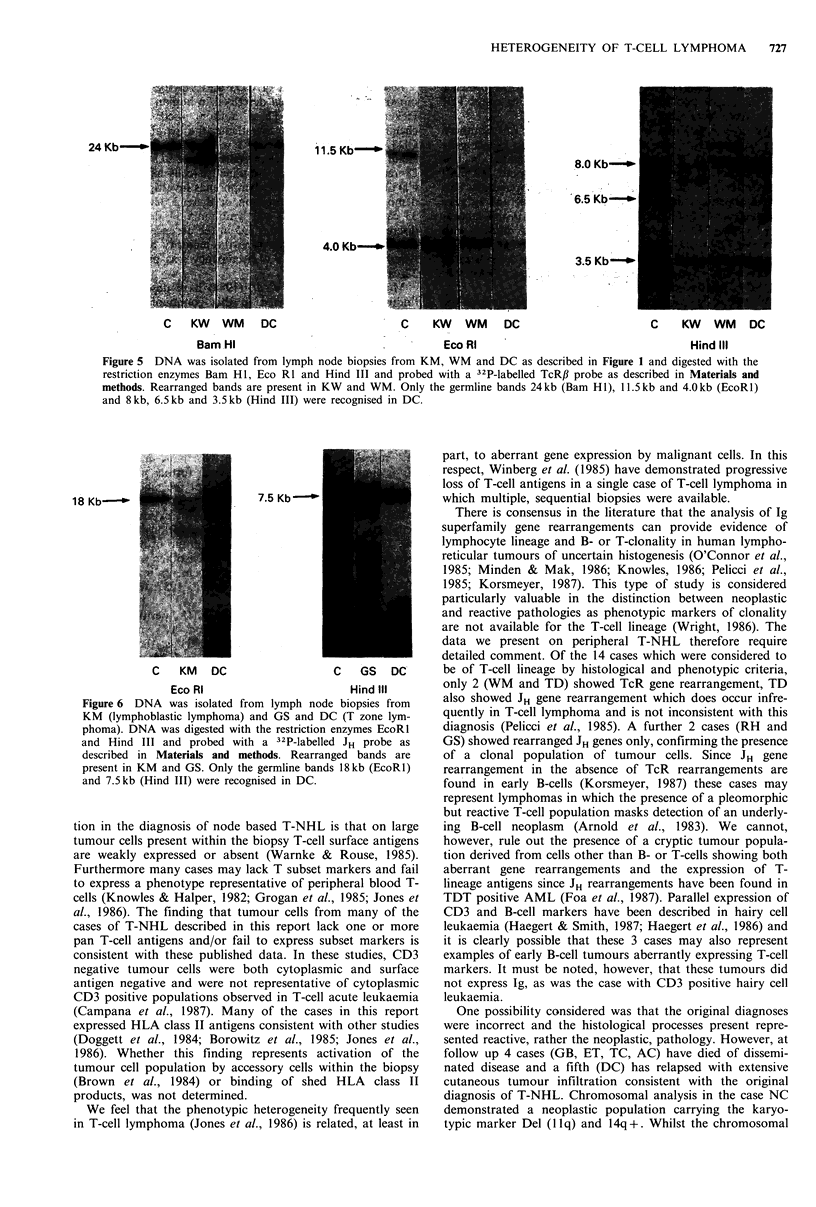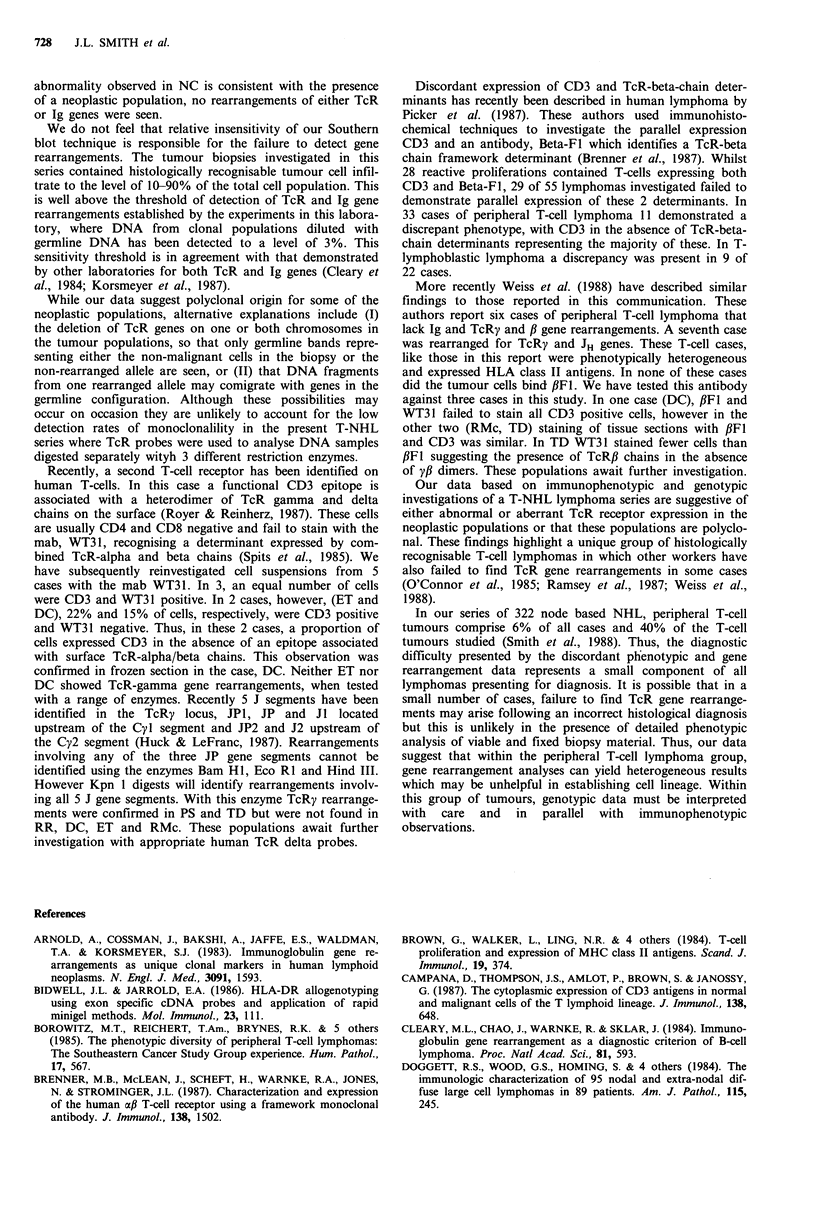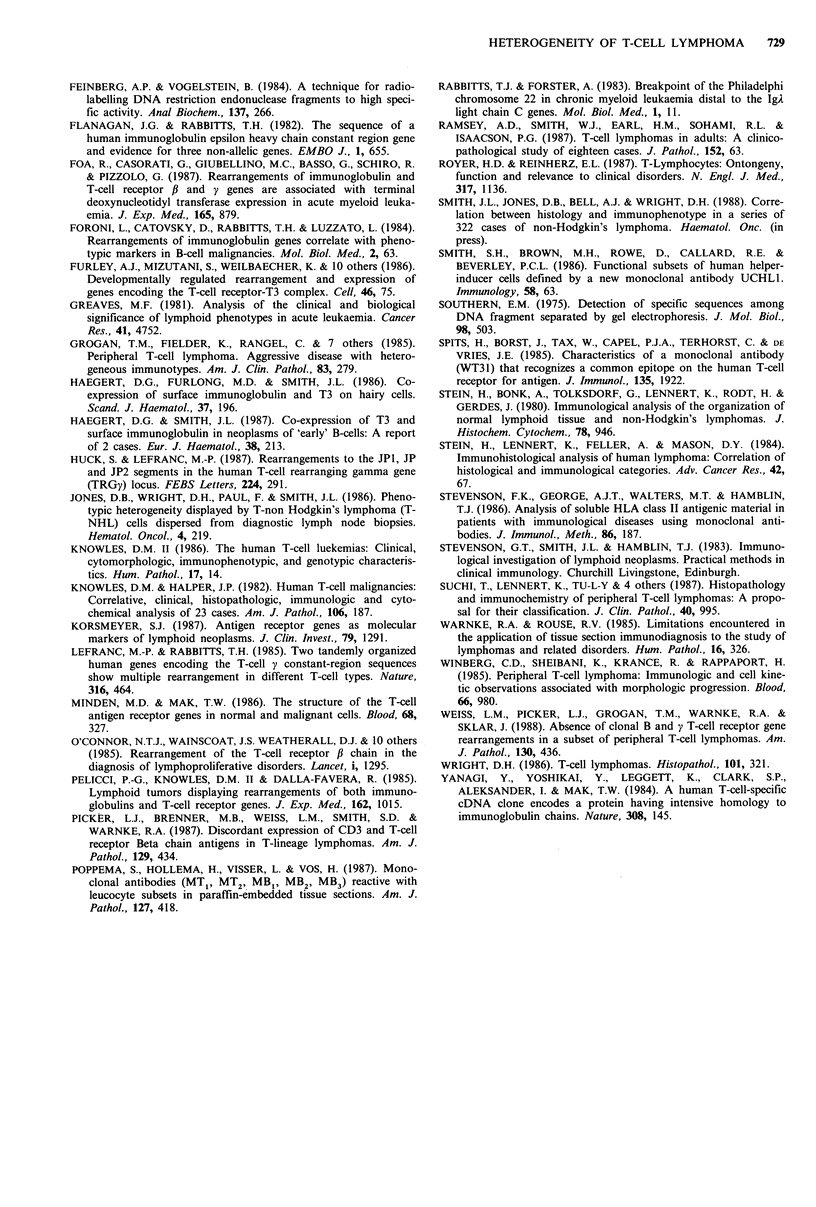# Phenotypic and genotypic heterogeneity of peripheral T-cell lymphoma.

**DOI:** 10.1038/bjc.1988.297

**Published:** 1988-12

**Authors:** J. L. Smith, D. G. Haegert, E. Hodges, G. N. Stacey, W. M. Howell, D. H. Wright, D. B. Jones

**Affiliations:** Regional Immunology Service, Southampton General Hospital.

## Abstract

**Images:**


Br. J. Cancer (1988), 58, 723–729 © The Macmillan Press Ltd., 1988

### Phenotypic and genotypic heterogeneity of peripheral T-cell lymphoma

J.L. Smith[1], D.G. Haegert[1], E. Hodges[1], G.N. Stacey[1], W.M. Howell[1], D.H. Wright[2] & D.B. Jones[2]

[1]*Regional Immunology Service; and* [2]*University Department of Pathology, Southampton General Hospital, Southampton.*

**Summary** A series of 21 phenotypically characterised T-cell lymphomas histologically defined as lymphocytic, lymphoblastic, immunoblastic, AILD type, pleomorphic, T-zone and Lennert's T-cell lymphoma, were investigated for T-cell receptor (TcR) and immunoglobulin (Ig) gene rearrangements. Phenotypic analyses of frozen sections and cell suspensions were heterogeneous and in many cases no single T-cell marker recognised all of the malignant cells. Data derived by staining with antibodies reactive with antigens in paraffin embedded tissue were consistent with T NHL in all cases except lymphoblastic lymphoma. TcR gene rearrangements were observed in lymphocytic, lymphoblastic and immunoblastic lymphoma, however, in the remaining 14 phenotypically and histologically defined peripheral T-cell lymphomas, 2 showed rearrangement of TcR$\gamma$ and $\beta$ genes consistent with T NHL and 2 showed Ig $J_H$ rearrangements only, suggestive of either reactive T-cell populations masking cryptic disease or presence of tumour populations with aberrant gene rearrangement and expression of T lineage antigens. No Ig or TcR gene rearrangements were found in the remaining 10 cases, in which morphologically identifiable tumour cells comprised 10–90% of the cell population. In 3/6 cases tested some CD3 positive cells failed to stain with WT31 or $\beta$F1, monoclonal antibodies that recognise determinants on combined TcR$\gamma\beta$ or TcR$\beta$ chains respectively. Whether these cases represent tumours arising from an undetermined cell of origin or polyclonal expansions of T-cells remains to be determined. Our results confirm the phenotypic heterogeneity of histologically defined peripheral T-cell lymphoma and indicate that in these particular histological subtypes gene rearrangement analysis can also yield heterogeneous results which may be unhelpful in determining cell lineage and clonality.

Phenotypic analyses have been widely used for the characterization of non-Hodgkin's lymphoma (NHL) and this approach has been most widely applied in NHL of B-cell origin (B-NHL) where surface phenotype can be closely correlated with the normal maturation pathway of immunoglobulin (Ig) producing lymphocytes (Stein *et al.*, 1980). The situation with NHL of T-cell lineage (T-NHL) is less clear. Whilst T-cell chronic lymphocytic leukaemia (T-CLL), mycosis fungoides and sezary syndrome regularly show a phenotype consistent with peripheral T-cells (Stein *et al.* 1984), the node-based T-NHL display great phenotypic heterogeneity (Jones *et al.*, 1986) and often represent a diagnostic difficulty. This is compounded by the absence of an immunocytochemical marker of monoclonality within the T-cell system (Wright, 1986). Analysis of T-cell receptor (TcR) gene rearrangement by Southern blot hybridization has become of considerable diagnostic importance in these difficult cases by providing evidence of clonality and of T-cell lineage. In this regard a number of investigators have found that the majority of T-cell leukaemias and lymphomas show T-cell receptor gene rearrangements (O'Connor *et al.*, 1985; Minden & Mak, 1986; Knowles, 1986; Pelicci *et al.*, 1985; Ramsey *et al.*, 1987). This has not been our experience in peripheral T-cell lymphomas.

Peripheral T-cell lymphomas represent a diverse histological group of neoplasms and heterogeneous phenotypes often with an admixture of cell types that make it difficult to recognize the neoplastic cell population (reviewed in Suchi *et al.*, 1987). In this study we report the results of an immunophenotypic and genotypic analysis of 17 cases of node based peripheral T-NHL, histologically characterised as histologies of AILD type, pleomorphic, T zone, Lennert's lymphoma, lymphocytic and immunoblastic lymphoma. These data are presented with findings on 4 cases of lymphoblastic lymphoma. Our results confirm that cases of peripheral T-NHL are markedly heterogeneous in phenotype and indicate that in histological subtypes other than lymphocytic and immunoblastic, TcR gene rearrangements are not always clonal. These data suggest that investigations of gene rearrangement within this group of tumours may often be unhelpful in establishing cell lineage and clonality.

Correspondence: J.L. Smith.
Received 8 April 1988; and in revised form, 25 July 1988.

### Materials and methods

### *Tissue*

Lymphoma tissue was obtained fresh from diagnostic lymph node biopsy specimens. Each specimen was subdivided and tissue taken for cell suspension preparation, histopathological diagnosis and for frozen section study. Lymphocytes were isolated from tissue by standard methods (Stevenson *et al.*, 1983) and the viability of the recovered cells was always greater than 95% by trypan blue exclusion. Lymphomas entered into this study were selected on the basis of their histological subtype and were drawn from a large series of lymphomas studied at Southampton (Smith *et al.*, 1988).

### *Phenotypic studies*

Phenotypic analyses were carried out on tissue sections and cell preparations using a panel of antibodies. Ig was demonstrated using rabbit anti-Ig heavy and light chain reagents (Dako) or with monoclonal antibodies (mabs) to k and $\lambda$ light chains. Lineage and subset markers were identified using the appropriate mabs identified by their workshop cluster numbers (Leucocyte Typing III, Oxford University Press, 1987) and detected by the corresponding mabs given in brackets, these included: CD1 (OKT6), CD2 (OKT11), CD3 (UCHT1, OKT3), CD4 (OKT4), CD5 (OKT1), CD7 ($HB_2$), CD8 (OKT8), CD22 (SCHL1), CD37 (WR17) and CD38 (OKT10). WR18, a mab recognising HLA DP, DQ and DR determinants, was also used (Stevenson *et al.*, 1986).

Biopsy cell suspensions from five cases were stained with the mab WT31 which recognises the TcR $\alpha\beta$ chains on the surface of T-cells (Spits *et al.*, 1985). Frozen biopsy sections from 3 cases were stained with WT31 and $\beta$F1, a mab which recognises a framework determinant on the TcR$\beta$ chain (Brenner *et al.*, 1987).

Cell suspensions were stained by indirect immunofluorescence; cytocentrifuge preparations were stained by indirect immunoperoxidase by methods previously described (Stevenson *et al.*, 1983). Immunohistochemical staining of frozen tissue was performed using a modification of the method of Stein *et al.* (1984). Briefly, 6 $\mu$m cryostat sections were dried at room temperature and stored at −20°C over dessicant until stained. On the day of staining sections were fixed in dry acetone for 15 min at room temperature and washed in Tris buffered saline, pH 7.6 (TBS). Mabs were then incubated with the sections for 30 min which were then washed twice in TBS. Peroxidase conjugated rabbit anti-mouse Ig (Dako, diluted 1/80 in TBS) was then applied for a further 30 min and the sections again washed.

Immunoperoxidase staining of paraffin embedded material was performed on 4 $\mu m$ sections using PAP or ABC complexes as previously described. Endogenous peroxidase activity was inhibited using 0.5% $H_2O_2$ in methanol for 10 min. The sections were pre-digested where necessary using 0.1% trypsin in 0.1% calcium chloride solution at 37°C. (Stevenson et al., 1983). All first layer staining reactions were incubated overnight at 4°C and appropriate controls performed for antibody specificity and titre. The following antibodies were used to stain paraffin sections: UCHL1 and MT1 (T-cell specific), MB2, 4KB5 (B-cell specific) and BER H2 (CD30 activated cells) (Poppema et al., 1987; Smith et al., 1986). For both paraffin and frozen sections peroxidase methods the final reaction product was developed using 3.3 diaminobenzidine tetrahydrochloride (DAB).

*Genotypic studies*

High molecular weight DNA was extracted from frozen biopsy specimens or cell suspensions by routine methods (Bidwell & Jarrold, 1986) and 10 $\mu g$ aliquots digested with 50 units of appropriate restriction endonucleases under conditions specified, DNA fragments were size-separated in 0.7% agarose gels and transferred to nylon (hybond-N) filters according to the method of Southern (1975). These filters were prehybridised in 6 x SSC, 0.5% SDS, 5 x Denhart's solution and 6% PEG 8,000 containing heterologous fragmented salmon sperm DNA at 65°C overnight, followed by hybridisation at 65°C overnight in the same solution containing added 32P-labelled gene probe. Filters were subsequently washed under stringent conditions (Bidwell & Jarrold, 1986) and hybridizing bands visualized by autoradiography for 3–10 days at −70°C, using Fuji-RX film. Following autoradiography, probe was removed from filters by washing with 0.4 M NaOH at 45° for 30 min followed by 0.1 x SSC, 0.1% SDS, 0.2 M Tris-HCl pH 7.5 at 45° for a further 30 min. Filters were then sequentially rehybridized with further gene probes.

The probes used in this study were an Ig heavy chain joining region $J_H$ probe (C76 R51A – Flanagan & Rabbitts, 1982), an Igk chain constant region probe (pUCR17 – Foroni et al., 1984) an Ig$\lambda$ constant region probe (CHR22$\lambda$S 8c$\lambda$ – Rabbitts & Forster, 1983), a TcR$\beta$ chain gene probe (Jurkat 2 – Yanagi et al., 1984) and a TcR$\gamma$ gene probe (Le Franc & Rabbitts, 1985).

Each DNA preparation was digested with 3 different restriction enzymes – Bam HI, Eco RI and Hind III. All digests were analysed with the TcR$\beta$ probe. The TcR$\gamma$ and $J_H$ probes were used to investigate at least two restriction digests per preparation, whilst the Ck probe was used in conjunction with Bam HI digests and C$\lambda$ with EcoRI digests. In addition, DNA from 7 cases was digested with the restriction enzyme KpnI and probed with the TcR$\gamma$ gene probe.

DNA probes were prepared by plasmid amplification in bacterial host stains. Probe gene inserts were excised from low gelling temperature agarose gels following appropriate endonuclease restriction and radiolabelled with $\alpha$-$^{32}$P-d CTP by the random hexanucleotide primer method (Feinberg & Vogelstein, 1984). In some experiments a TcR$\beta$ gene probe of very high specific activity was produced by labelling with two radionucleotides instead of one ($\alpha$-$^{32}$P-dATP and $\alpha$-$^{32}$P-dATP).

The sensitivity of the TcR$\gamma$ and $\beta$ chain gene probing was assessed by dilution of DNA isolated from a T-cell immunoblastic lymphoma (PB) with germline DNA isolated from a fresh carcinoma biopsy followed by Southern hybridization. The sensitivity of the $J_H$ and Ck immunoglobulin gene probing was similarly assessed by dilution of DNA isolated from a lymphoid cell suspension containing >99% surface immunoglobulin Mk positive cells prepared from the blood of a patient with B-cell chronic lymphocytic leukaemia.

### Results

*Histology and immunophenotype*

Fourteen cases of morphologically defined T-cell NHL entered into the study were given given the following diagnoses based on initial histology: AILD type (WM, RH), pleomorphic T-cell (large type) (RR), pleomorphic T-cell (medium type) (NC), T zone (TD, GS, DC, BM, ET, GB, JR, TC, AC) and Lennert's NHL (RMc). Seven cases of phenotypically defined T-cell lymphoma of the following histologies, lymphoblastic (RQ, KM, PSh, KW), immunoblastic (PS, PB) and lymphocytic (WS) were also studied. In all biopsies the percentage of neoplastic cells was assessed morphologically on paraffin embedded material stained with haematoxilin and eosin (H and E). Phenotypic characterisation was performed in cell suspension and in frozen and paraffin sections. The results of frozen section and cell suspension analyses were closely concordant and in 18 cases the data were consistent with T-NHL: in the remaining three cases (ET, JR, RMc) the lineage of the neoplastic cell population could not be determined unequivocally from these data. Data derived from paraffin sections were consistent with T-NHL in 17 cases; in the remaining 4 cases, neoplastic cell staining was observed with UCHL1 and MB2 in case NC and with MB2 in cases RQ, KM and PSh. All neoplastic cells in preparations were Ig negative and failed to stain with B-cell antibodies identifying CD22 and CD37 pan B-cell antigens. A summary of the phenotypic data is given in Table I and two cases are illustrated, WM (AILD type) in Figure 1 and DC (T zone) in Figure 2.

In the data presented in Table I, atypical neoplastic cells from 10 cases in frozen tissue sections and in cell preparations were either variably stained (WM, RH, TD, BM, NC, RR, PS) or were negative (AC, PB, PSh) for CD3. The CD3 negative cells were cytoplasmic CD3 negative on staining of frozen sections and cytocentrifuge preparations. This variability was less evident with CD2 but CD7 was variably expressed in 2 cases (WM, TD) and was absent in 4 cases (AC, NC, RR, PS). Not all cases were stained with CD4 and CD8 antibodies, however CD8 was present in 3 cases in the absence of CD4 (RH, GB, TC) and CD4 was present in the absence of CD8 in 4 cases (DC, WS, RQ, KM). Neoplastic cells from 5 cases did not express CD4 or CD8. Cells from 8 cases expressed HLA class II antigens (Table I).

Cell suspensions from 5 cases (WM, DC, ET, GB and NC) were stained with the mab WT31 which recognises TcR $\alpha\beta$ chains on the surface of T-cells. (Spits et al., 1985). In 3 cases staining with CD3 and WT31 was in agreement, however in 2 (DC and ET) WT31 failed to stain 22% and 15% of the CD3+ cells in the preparations respectively (CD3 stained 77% and 49% of cells in DC and ET preparations respectively). WT31 and $\beta$F1 mabs were used to stain frozen biopsy sections from DC, TD and RMc. In DC WT31 and $\beta$F1 stained fewer cells than those positive for CD3. In RMc the number of CD3, WT31 and $\beta$F1 were similar. In TD the number of CD3 and $\beta$F1 positive cells were similar and greater than the number of cells stained by the mab WT31.

*Gene rearrangements*

The sensitivity of the TcR$\gamma$ and $\beta$ chain gene probing was investigated by dilution of tumour DNA from case PB (immunoblastic lymphoma) with germline DNA. TcR$\gamma$ and $\beta$ gene rearrangements were readily detectable when tumour DNA represented 3% of the total DNA analysed. The TcR$\beta$ probing is illustrated in Figure 3. Similarly $J_H$ and Ck Ig gene rearrangements were detectable at dilutions of 3% of B-cell tumour DNA with germline DNA. The four cases of T

**Table I** Neoplastic cell phenotype

| Histology | Case | Neoplastic cells % | CD1 | CD2 | CD3 | CD4 | CD5 | CD7 | CD8 | CD38 | HLA class II | UCHL1 | MT1 | BERH2 | MB2 | 4KB5 |
|---|---|---|---|---|---|---|---|---|---|---|---|---|---|---|---|---|
| | | | *Frozen section/cell suspensions*[a] | | | | | | | | | *Paraffin sections*[a] | | | | |
| AILD type | WM | 40 | + | + | (+) | | + | (+) | (+) | + | + | + | + | – | – | – |
| | RH | 30 | – | + | (+) | – | + | | + | + | | + | + | + | – | – |
| T zone | TD | 30 | | + | (+) | | + | (+) | – | | + | + | + | – | – | – |
| | GS | 70 | | + | + | – | | + | – | | – | + | + | – | – | – |
| | DC | 30 | – | + | + | + | + | + | – | + | – | + | + | + | – | – |
| | BM | 30 | | | (+) | | + | + | – | | + | + | – | – | – | – |
| | ET | 60[b] | | | | | | | | | | (+) | + | – | – | – |
| | GB | 50 | – | + | + | – | + | + | + | – | + | + | + | – | – | – |
| | JR | 50[b] | | | | | | | | | | + | + | – | – | – |
| | TC | 50 | + | + | + | – | + | + | + | + | + | + | + | – | – | – |
| | AC | 50 | – | + | – | – | + | – | – | + | – | + | + | – | – | – |
| Lennert's | RMc | 25[b] | | | | | | | | | | + | + | – | – | – |
| Pleomorphic medium | NC | 90 | (+) | (+) | (+) | – | – | – | – | + | + | + | – | – | + | – |
| Pleomorphic large | RR | 10 | | + | (+) | | | – | – | | + | + | + | – | – | – |
| Immunoblastic | PS | 80 | (+) | + | (+) | – | – | – | – | | – | + | + | – | – | – |
| | PB | 98 | – | + | – | | | | – | | + | + | – | + | – | – |
| Lymphocytic | WS | 90 | | | + | + | + | + | – | | – | + | + | – | – | – |
| Lymphoblastic | RQ | 85 | + | (+) | + | + | | + | – | | – | – | – | – | + | – |
| | KM | 90 | | + | + | (+) | + | + | – | | – | – | – | – | + | – |
| | PSh | 80 | + | + | – | – | + | + | – | + | – | – | – | – | + | – |
| | KW | 50 | | + | + | + | | | | | – | + | + | – | – | – |

Blanks in table refer to tests not done. [a]+ positive. (+) variable staining on tumour cell population, – negative. All neoplastic cells were negative for Ig, CD22 and CD37. % of neoplastic cells in biopsies on sections of paraffin embedded tissue stained with H and E. [b]Neoplastic cell phenotype could not be adequately defined on frozen section or in cell suspension.

**Figure 1** (a-UCHL1, b-MT1) WM (T-Cell Receptor Gene Rearranged): Sections of this case stained with paraffin reactive T-cell antibodies, UCHL1 and MT1. There is extensive staining of the tumour population with many morphologically identifiable tumour cells staining.

**Figure 2** (a-UCHL1, b-MT1) DC (T-Cell Receptor Germline): The T-cell directed antibodies, UCHL1 and MT1, stain the bulk of the infiltrating cells in this lymph node biopsy. Large, morphologically identifiable tumour cells are positive with both reagents. This appearance in paraffin is consistent with T-cell lymphoma.

**Figure 3** DNA was isolated from lymph node biopsy PB (immunoblastic lymphoma) and diluted with germline DNA isolated from a carcinoma. DNA was digested with Hind III and probed with a $^{32}P$ labelled TcR$\beta$ probe as described in the methods. Germline bands of 6.5 and 3.5 kb were detected, reflecting complete DNA digestion by Hind III. No 8.0 kb band, which results from partial cutting at one Hind III site, was seen (Furley *et al*., 1986). The rearranged band is detectable at 3% dilution of tumour DNA.

**Table II** Genotype of peripheral T lymphomas

| *Histology* | *Case*[b] | *Gene rearrangement*[a] *TcR* $\gamma$ | *TcR* $\beta$ | *Ig* $J_H$ | *Ig* $C_k$ | *Ig* $C_\lambda$ |
|---|---|---|---|---|---|---|
| AILD type | WM | R | R | G | G | G |
| | RH | G | G | R | G | |
| T zone | TD | R | R | R | G | G |
| | GS | G | G | R | G | G |
| | DC, BM, ET GB, JR, TC AC | G | G | G | G | G |
| Pleomorphic medium | NC | G | G | G | G | G |
| Pleomorphic large | RR | G | G | G | G | G |
| Lennert's | RMc | G | G | G | G | G |
| Immunoblastic | PS | R | R | G | G | |
| | PB | R | R | G | G | G |
| Lymphocytic | WS | | R | G | G | |
| Lymphoblastic | RQ | R | R | G | G | G |
| | KM | R | R | R | G | G |
| | PSh | R | R | G | G | |
| | KW | R | R | G | G | |

Blanks in table refer to tests not done. [a]R clonal rearrangement; G germline configuration. [b]DNA for analysis was isolated from frozen biopsy tissue from cases WM, RH, TD, GS, DC, JR, RMc, NC, RR, KM, PS, PB & WS. Remaining cases BM, ET, GB, TC, AC, RQ, PSh & KW DNA was isolated from cell suspensions prepared from fresh biopsy tissue.

lymphoblastic lymphoma all showed TcR$\gamma$ and $\beta$ gene rearrangement, in addition one of the cases (KM) also show $J_H$ gene rearrangement. In these cases the percentage of tumour cells varied from 50–90% (Table II).

Southern hybridization analyses of the peripheral T-cell lymphomas revealed TcR gene rearrangements in the lymphocytic (WS), immunoblastic lymphomas (PS, PB) and in one case of AILD type (WM) and in one case of T zone NHL (TD) (Table II); TD also showed $J_H$ gene rearrangement. A further two cases (RH, GS) showed $J_H$ gene rearrangements only. The remaining 10 cases showed no detectable TcR or Ig gene rearrangements. These data are summarized in Table II. Representative data are shown in Figures 4–6.

**Figure 4** DNA was isolated from lymph node biopsies from PS (immunoblastic lymphoma) PSh, KW (T lymphoblastic lymphoma) WM (AILD lymphoma) and DC (T zone lymphoma). Control DNA was isolated from a lymph node biopsy involved with carcinoma. DNA was digested with the restriction enzyme Bam H1 and probed with a $^{32}P$-labelled TcR$\gamma$ probe as described in **Materials and methods**. Rearranged bands are present in PS, PSh, KW and WM. Only the germline bands at 15 and 12.5 kb were recognised in DC.

TcR$\beta$ gene probing with probe labelled with two nucleotides (see **Materials and methods**) was carried out on DNA from 4 patients but failed to reveal TcR$\beta$ gene rearrangements in RR, GS, DC or ET. Kpn 1 enzyme digests were completed on DNA from RR, PS, DC, TD, ET, TC and RMc and analysed with the TcR$\gamma$ gene probe. TcR$\gamma$ gene rearrangements were confirmed in PS and TD but were not seen in the other cases tested.

### Discussion

T-NHL is often associated with a characteristic histological picture. However, the association is not absolute and a firm diagnosis often requires phenotypic confirmation (Wright, 1986). All the cases of lymphoblastic, immunoblastic and lymphocytic lymphoma were shown to be of T-cell phenotype by the expression of one or more pan T-cell antigens. Eleven of the 14 cases presented in this communication and considered to be of T-cell origin on morphological criteria alone, demonstrated a phenotypic pattern consistent with a diagnosis of T-NHL when examined with monoclonal antibodies recognising the T-cell lineage associated markers, CD2, CD3 and CD7. In the remaining 3 cases the precise surface phenotype of the tumour cells could not be determined in cell suspension or in frozen section. In paraffin section the antibodies MT1 and/or UCHL1 stained tumour cells in 18 cases including all cases of peripheral T-cell lymphoma; MB2 stained tumour cells in three cases of lymphoblastic (RQ, KM, PSh) and in one case (NC) of pleomorphic lymphoma, these data are consistent with published findings which report that staining with these antibodies is not an absolute marker for the T- or B-cell lineage (Poppema *et al*., 1987).

Phenotypic variation is a common feature amongst T-cell lymphomas and whilst leukaemias derived from early T-cells may display consistent phenotypic features (Greaves, 1981) the general picture for node based T-cell lymphoma is one of heterogeneity (Jones *et al*., 1986). An additional complica-

**Figure 5** DNA was isolated from lymph node biopsies from KM, WM and DC as described in **Figure 1** and digested with the restriction enzymes Bam H1, Eco R1 and Hind III and probed with a $^{32}$P-labelled TcR$\beta$ probe as described in **Materials and methods**. Rearranged bands are present in KW and WM. Only the germline bands 24 kb (Bam H1), 11.5 kb and 4.0 kb (EcoR1) and 8 kb, 6.5 kb and 3.5 kb (Hind III) were recognised in DC.

**Figure 6** DNA was isolated from lymph node biopsies from KM (lymphoblastic lymphoma) and GS and DC (T zone lymphoma). DNA was digested with the restriction enzymes EcoR1 and Hind III and probed with a $^{32}$P-labelled $J_H$ probe as described in **Materials and methods**. Rearranged bands are present in KM and GS. Only the germline bands 18 kb (EcoR1) and 7.5 kb (Hind III) were recognised in DC.

tion in the diagnosis of node based T-NHL is that on large tumour cells present within the biopsy T-cell surface antigens are weakly expressed or absent (Warnke & Rouse, 1985). Furthermore many cases may lack T subset markers and fail to express a phenotype representative of peripheral blood T-cells (Knowles & Halper, 1982; Grogan *et al.*, 1985; Jones *et al.*, 1986). The finding that tumour cells from many of the cases of T-NHL described in this report lack one or more pan T-cell antigens and/or fail to express subset markers is consistent with these published data. In these studies, CD3 negative tumour cells were both cytoplasmic and surface antigen negative and were not representative of cytoplasmic CD3 positive populations observed in T-cell acute leukaemia (Campana *et al.*, 1987). Many of the cases in this report expressed HLA class II antigens consistent with other studies (Doggett *et al.*, 1984; Borowitz *et al.*, 1985; Jones *et al.*, 1986). Whether this finding represents activation of the tumour cell population by accessory cells within the biopsy (Brown *et al.*, 1984) or binding of shed HLA class II products, was not determined.

We feel that the phenotypic heterogeneity frequently seen in T-cell lymphoma (Jones *et al.*, 1986) is related, at least in part, to aberrant gene expression by malignant cells. In this respect, Winberg *et al.* (1985) have demonstrated progressive loss of T-cell antigens in a single case of T-cell lymphoma in which multiple, sequential biopsies were available.

There is consensus in the literature that the analysis of Ig superfamily gene rearrangements can provide evidence of lymphocyte lineage and B- or T-clonality in human lymphoreticular tumours of uncertain histogenesis (O'Connor *et al.*, 1985; Minden & Mak, 1986; Knowles, 1986; Pelicci *et al.*, 1985; Korsmeyer, 1987). This type of study is considered particularly valuable in the distinction between neoplastic and reactive pathologies as phenotypic markers of clonality are not available for the T-cell lineage (Wright, 1986). The data we present on peripheral T-NHL therefore require detailed comment. Of the 14 cases which were considered to be of T-cell lineage by histological and phenotypic criteria, only 2 (WM and TD) showed TcR gene rearrangement, TD also showed $J_H$ gene rearrangement which does occur infrequently in T-cell lymphoma and is not inconsistent with this diagnosis (Pelicci *et al.*, 1985). A further 2 cases (RH and GS) showed rearranged $J_H$ genes only, confirming the presence of a clonal population of tumour cells. Since $J_H$ gene rearrangement in the absence of TcR rearrangements are found in early B-cells (Korsmeyer, 1987) these cases may represent lymphomas in which the presence of a pleomorphic but reactive T-cell population masks detection of an underlying B-cell neoplasm (Arnold *et al.*, 1983). We cannot, however, rule out the presence of a cryptic tumour population derived from cells other than B- or T-cells showing both aberrant gene rearrangements and the expression of T-lineage antigens since $J_H$ rearrangements have been found in TDT positive AML (Foa *et al.*, 1987). Parallel expression of CD3 and B-cell markers have been described in hairy cell leukaemia (Haegert & Smith, 1987; Haegert *et al.*, 1986) and it is clearly possible that these 3 cases may also represent examples of early B-cell tumours aberrantly expressing T-cell markers. It must be noted, however, that these tumours did not express Ig, as was the case with CD3 positive hairy cell leukaemia.

One possibility considered was that the original diagnoses were incorrect and the histological processes present represented reactive, rather the neoplastic, pathology. However, at follow up 4 cases (GB, ET, TC, AC) have died of disseminated disease and a fifth (DC) has relapsed with extensive cutaneous tumour infiltration consistent with the original diagnosis of T-NHL. Chromosomal analysis in the case NC demonstrated a neoplastic population carrying the karyotypic marker Del (11q) and 14q+. Whilst the chromosomal abnormality observed in NC is consistent with the presence of a neoplastic population, no rearrangements of either TcR or Ig genes were seen.

We do not feel that relative insensitivity of our Southern blot technique is responsible for the failure to detect gene rearrangements. The tumour biopsies investigated in this series contained histologically recognisable tumour cell infiltrate to the level of 10–90% of the total cell population. This is well above the threshold of detection of TcR and Ig gene rearrangements established by the experiments in this laboratory, where DNA from clonal populations diluted with germline DNA has been detected to a level of 3%. This sensitivity threshold is in agreement with that demonstrated by other laboratories for both TcR and Ig genes (Cleary *et al.*, 1984; Korsmeyer *et al.*, 1987).

While our data suggest polyclonal origin for some of the neoplastic populations, alternative explanations include (I) the deletion of TcR genes on one or both chromosomes in the tumour populations, so that only germline bands representing either the non-malignant cells in the biopsy or the non-rearranged allele are seen, or (II) that DNA fragments from one rearranged allele may comigrate with genes in the germline configuration. Although these possibilities may occur on occasion they are unlikely to account for the low detection rates of monoclonalility in the present T-NHL series where TcR probes were used to analyse DNA samples digested separately wityh 3 different restriction enzymes.

Recently, a second T-cell receptor has been identified on human T-cells. In this case a functional CD3 epitope is associated with a heterodimer of TcR gamma and delta chains on the surface (Royer & Reinherz, 1987). These cells are usually CD4 and CD8 negative and fail to stain with the mab, WT31, recognising a determinant expressed by combined TcR-alpha and beta chains (Spits *et al.*, 1985). We have subsequently reinvestigated cell suspensions from 5 cases with the mab WT31. In 3, an equal number of cells were CD3 and WT31 positive. In 2 cases, however, (ET and DC), 22% and 15% of cells, respectively, were CD3 positive and WT31 negative. Thus, in these 2 cases, a proportion of cells expressed CD3 in the absence of an epitope associated with surface TcR-alpha/beta chains. This observation was confirmed in frozen section in the case, DC. Neither ET nor DC showed TcR-gamma gene rearrangements, when tested with a range of enzymes. Recently 5 J segments have been identified in the TcR$\gamma$ locus, JP1, JP and J1 located upstream of the C$\gamma$1 segment and JP2 and J2 upstream of the C$\gamma$2 segment (Huck & LeFranc, 1987). Rearrangements involving any of the three JP gene segments cannot be identified using the enzymes Bam H1, Eco R1 and Hind III. However Kpn 1 digests will identify rearrangements involving all 5 J gene segments. With this enzyme TcR$\gamma$ rearrangements were confirmed in PS and TD but were not found in RR, DC, ET and RMc. These populations await further investigation with appropriate human TcR delta probes.

Discordant expression of CD3 and TcR-beta-chain determinants has recently been described in human lymphoma by Picker *et al.* (1987). These authors used immunohistochemical techniques to investigate the parallel expression CD3 and an antibody, Beta-F1 which identifies a TcR-beta chain framework determinant (Brenner *et al.*, 1987). Whilst 28 reactive proliferations contained T-cells expressing both CD3 and Beta-F1, 29 of 55 lymphomas investigated failed to demonstrate parallel expression of these 2 determinants. In 33 cases of peripheral T-cell lymphoma 11 demonstrated a discrepant phenotype, with CD3 in the absence of TcR-beta-chain determinants representing the majority of these. In T-lymphoblastic lymphoma a discrepancy was present in 9 of 22 cases.

More recently Weiss *et al.* (1988) have described similar findings to those reported in this communication. These authors report six cases of peripheral T-cell lymphoma that lack Ig and TcR$\gamma$ and $\beta$ gene rearrangements. A seventh case was rearranged for TcR$\gamma$ and $J_H$ genes. These T-cell cases, like those in this report were phenotypically heterogeneous and expressed HLA class II antigens. In none of these cases did the tumour cells bind $\beta$F1. We have tested this antibody against three cases in this study. In one case (DC), $\beta$F1 and WT31 failed to stain all CD3 positive cells, however in the other two (RMc, TD) staining of tissue sections with $\beta$F1 and CD3 was similar. In TD WT31 stained fewer cells than $\beta$F1 suggesting the presence of TcR$\beta$ chains in the absence of $\gamma\beta$ dimers. These populations await further investigation.

Our data based on immunophenotypic and genotypic investigations of a T-NHL lymphoma series are suggestive of either abnormal or aberrant TcR receptor expression in the neoplastic populations or that these populations are polyclonal. These findings highlight a unique group of histologically recognisable T-cell lymphomas in which other workers have also failed to find TcR gene rearrangements in some cases (O'Connor *et al.*, 1985; Ramsey *et al.*, 1987; Weiss *et al.*, 1988).

In our series of 322 node based NHL, peripheral T-cell tumours comprise 6% of all cases and 40% of the T-cell tumours studied (Smith *et al.*, 1988). Thus, the diagnostic difficulty presented by the discordant phenotypic and gene rearrangement data represents a small component of all lymphomas presenting for diagnosis. It is possible that in a small number of cases, failure to find TcR gene rearrangements may arise following an incorrect histological diagnosis but this is unlikely in the presence of detailed phenotypic analysis of viable and fixed biopsy material. Thus, our data suggest that within the peripheral T-cell lymphoma group, gene rearrangement analyses can yield heterogeneous results which may be unhelpful in establishing cell lineage. Within this group of tumours, genotypic data must be interpreted with care and in parallel with immunophenotypic observations.

### References

ARNOLD, A., COSSMAN, J., BAKSHI, A., JAFFE, E.S., WALDMAN, T.A. & KORSMEYER, S.J. (1983). Immunoglobulin gene rearrangements as unique clonal markers in human lymphoid neoplasms. *N. Engl. J. Med.*, **3091**, 1593.

BIDWELL, J.L. & JARROLD, E.A. (1986). HLA-DR allogenotyping using exon specific cDNA probes and application of rapid minigel methods. *Mol. Immunol.*, **23**, 111.

BOROWITZ, M.T., REICHERT, T.Am., BRYNES, R.K. & 5 others (1985). The phenotypic diversity of peripheral T-cell lymphomas: The Southeastern Cancer Study Group experience. *Hum. Pathol.*, **17**, 567.

BRENNER, M.B., McLEAN, J., SCHEFT, H., WARNKE, R.A., JONES, N. & STROMINGER, J.L. (1987). Characterization and expression of the human $\alpha\beta$ T-cell receptor using a framework monoclonal antibody. *J. Immunol.*, **138**, 1502.

BROWN, G., WALKER, L., LING, N.R. & 4 others (1984). T-cell proliferation and expression of MHC class II antigens. *Scand. J. Immunol.*, **19**, 374.

CAMPANA, D., THOMPSON, J.S., AMLOT, P., BROWN, S. & JANOSSY, G. (1987). The cytoplasmic expression of CD3 antigens in normal and malignant cells of the T lymphoid lineage. *J. Immunol.*, **138**, 648.

CLEARY, M.L., CHAO, J., WARNKE, R. & SKLAR, J. (1984). Immunoglobulin gene rearrangement as a diagnostic criterion of B-cell lymphoma. *Proc. Natl Acad. Sci.*, **81**, 593.

DOGGETT, R.S., WOOD, G.S., HOMING, S. & 4 others (1984). The immunologic characterization of 95 nodal and extra-nodal diffuse large cell lymphomas in 89 patients. *Am. J. Pathol.*, **115**, 245.

FEINBERG, A.P. & VOGELSTEIN, B. (1984). A technique for radiolabelling DNA restriction endonuclease fragments to high specific activity. *Anal Biochem.*, **137**, 266.

FLANAGAN, J.G. & RABBITTS, T.H. (1982). The sequence of a human immunoglobulin epsilon heavy chain constant region gene and evidence for three non-allelic genes. *EMBO J.*, **1**, 655.

FOA, R., CASORATI, G., GIUBELLINO, M.C., BASSO, G., SCHIRO, R. & PIZZOLO, G. (1987). Rearrangements of immunoglobulin and T-cell receptor $\beta$ and $\gamma$ genes are associated with terminal deoxynucleotidyl transferase expression in acute myeloid leukaemia. *J. Exp. Med.*, **165**, 879.

FORONI, L., CATOVSKY, D., RABBITTS, T.H. & LUZZATO, L. (1984). Rearrangements of immunoglobulin genes correlate with phenotypic markers in B-cell malignancies. *Mol. Biol. Med.*, **2**, 63.

FURLEY, A.J., MIZUTANI, S., WEILBAECHER, K. & 10 others (1986). Developmentally regulated rearrangement and expression of genes encoding the T-cell receptor-T3 complex. *Cell*, **46**, 75.

GREAVES, M.F. (1981). Analysis of the clinical and biological significance of lymphoid phenotypes in acute leukaemia. *Cancer Res.*, **41**, 4752.

GROGAN, T.M., FIELDER, K., RANGEL, C. & 7 others (1985). Peripheral T-cell lymphoma. Aggressive disease with heterogeneous immunotypes. *Am. J. Clin. Pathol.*, **83**, 279.

HAEGERT, D.G., FURLONG, M.D. & SMITH, J.L. (1986). Co-expression of surface immunoglobulin and T3 on hairy cells. *Scand. J. Haematol.*, **37**, 196.

HAEGERT, D.G. & SMITH, J.L. (1987). Co-expression of T3 and surface immunoglobulin in neoplasms of 'early' B-cells: A report of 2 cases. *Eur. J. Haematol.*, **38**, 213.

HUCK, S. & LEFRANC, M.-P. (1987). Rearrangements to the JP1, JP and JP2 segments in the human T-cell rearranging gamma gene (TRG$\gamma$) locus. *FEBS Letters*, **224**, 291.

JONES, D.B., WRIGHT, D.H., PAUL, F. & SMITH, J.L. (1986). Phenotypic heterogeneity displayed by T-non Hodgkin's lymphoma (T-NHL) cells dispersed from diagnostic lymph node biopsies. *Hematol. Oncol.*, **4**, 219.

KNOWLES, D.M. II (1986). The human T-cell luekemias: Clinical, cytomorphologic, immunophenotypic, and genotypic characteristics. *Hum. Pathol.*, **17**, 14.

KNOWLES, D.M. & HALPER, J.P. (1982). Human T-cell malignancies: Correlative, clinical, histopathologic, immunologic and cytochemical analysis of 23 cases. *Am. J. Pathol.*, **106**, 187.

KORSMEYER, S.J. (1987). Antigen receptor genes as molecular markers of lymphoid neoplasms. *J. Clin. Invest.*, **79**, 1291.

LEFRANC, M.-P. & RABBITTS, T.H. (1985). Two tandemly organized human genes encoding the T-cell $\gamma$ constant-region sequences show multiple rearrangement in different T-cell types. *Nature*, **316**, 464.

MINDEN, M.D. & MAK, T.W. (1986). The structure of the T-cell antigen receptor genes in normal and malignant cells. *Blood*, **68**, 327.

O'CONNOR, N.T.J., WAINSCOAT, J.S. WEATHERALL, D.J. & 10 others (1985). Rearrangement of the T-cell receptor $\beta$ chain in the diagnosis of lymphoproliferative disorders. *Lancet*, **i**, 1295.

PELICCI, P.-G., KNOWLES, D.M. II & DALLA-FAVERA, R. (1985). Lymphoid tumors displaying rearrangements of both immunoglobulins and T-cell receptor genes. *J. Exp. Med.*, **162**, 1015.

PICKER, L.J., BRENNER, M.B., WEISS, L.M., SMITH, S.D. & WARNKE, R.A. (1987). Discordant expression of CD3 and T-cell receptor Beta chain antigens in T-lineage lymphomas. *Am. J. Pathol.*, **129**, 434.

POPPEMA, S., HOLLEMA, H., VISSER, L. & VOS, H. (1987). Monoclonal antibodies ($MT_1$, $MT_2$, $MB_1$, $MB_2$, $MB_3$) reactive with leucocyte subsets in paraffin-embedded tissue sections. *Am. J. Pathol.*, **127**, 418.

RABBITTS, T.J. & FORSTER, A. (1983). Breakpoint of the Philadelphi chromosome 22 in chronic myeloid leukaemia distal to the Ig$\lambda$ light chain C genes. *Mol. Biol. Med.*, **1**, 11.

RAMSEY, A.D., SMITH, W.J., EARL, H.M., SOHAMI, R.L. & ISAACSON, P.G. (1987). T-cell lymphomas in adults: A clinicopathological study of eighteen cases. *J. Pathol.*, **152**, 63.

ROYER, H.D. & REINHERZ, E.L. (1987). T-Lymphocytes: Ontongeny, function and relevance to clinical disorders. *N. Engl. J. Med.*, **317**, 1136.

SMITH, J.L., JONES, D.B., BELL, A.J. & WRIGHT, D.H. (1988). Correlation between histology and immunophenotype in a series of 322 cases of non-Hodgkin's lymphoma. *Haematol. Onc.* (in press).

SMITH, S.H., BROWN, M.H., ROWE, D., CALLARD, R.E. & BEVERLEY, P.C.L. (1986). Functional subsets of human helper-inducer cells defined by a new monoclonal antibody UCHL1. *Immunology*, **58**, 63.

SOUTHERN, E.M. (1975). Detection of specific sequences among DNA fragment separated by gel electrophoresis. *J. Mol. Biol.*, **98**, 503.

SPITS, H., BORST, J., TAX, W., CAPEL, P.J.A., TERHORST, C. & DE VRIES, J.E. (1985). Characteristics of a monoclonal antibody (WT31) that recognizes a common epitope on the human T-cell receptor for antigen. *J. Immunol.*, **135**, 1922.

STEIN, H., BONK, A., TOLKSDORF, G., LENNERT, K., RODT, H. & GERDES, J. (1980). Immunological analysis of the organization of normal lymphoid tissue and non-Hodgkin's lymphomas. *J. Histochem. Cytochem.*, **78**, 946.

STEIN, H., LENNERT, K., FELLER, A. & MASON, D.Y. (1984). Immunohistological analysis of human lymphoma: Correlation of histological and immunological categories. *Adv. Cancer Res.*, **42**, 67.

STEVENSON, F.K., GEORGE, A.J.T., WALTERS, M.T. & HAMBLIN, T.J. (1986). Analysis of soluble HLA class II antigenic material in patients with immunological diseases using monoclonal antibodies. *J. Immunol., Meth.*, **86**, 187.

STEVENSON, G.T., SMITH, J.L. & HAMBLIN, T.J. (1983). Immunological investigation of lymphoid neoplasms. Practical methods in clinical immunology. Churchill Livingstone, Edinburgh.

SUCHI, T., LENNERT, K., TU-L-Y & 4 others (1987). Histopathology and immunochemistry of peripheral T-cell lymphomas: A proposal for their classification. *J. Clin. Pathol.*, **40**, 995.

WARNKE, R.A. & ROUSE, R.V. (1985). Limitations encountered in the application of tissue section immunodiagnosis to the study of lymphomas and related disorders. *Hum. Pathol.*, **16**, 326.

WINBERG, C.D., SHEIBANI, K., KRANCE, R. & RAPPAPORT, H. (1985). Peripheral T-cell lymphoma: Immunologic and cell kinetic observations associated with morphologic progression. *Blood*, **66**, 980.

WEISS, L.M., PICKER, L.J., GROGAN, T.M., WARNKE, R.A. & SKLAR, J. (1988). Absence of clonal B and $\gamma$ T-cell receptor gene rearrangements in a subset of peripheral T-cell lymphomas. *Am. J. Pathol.*, **130**, 436.

WRIGHT, D.H. (1986). T-cell lymphomas. *Histopathol.*, **101**, 321.

YANAGI, Y., YOSHIKAI, Y., LEGGETT, K., CLARK, S.P., ALEKSANDER, I. & MAK, T.W. (1984). A human T-cell-specific cDNA clone encodes a protein having intensive homology to immunoglobulin chains. *Nature*, **308**, 145.